# Anton syndrome

**DOI:** 10.1007/s10072-022-06475-0

**Published:** 2022-11-07

**Authors:** Marina Romozzi, Gabriele Lucioli, Sofia Marini, Mauro Monforte, Aldobrando Broccolini

**Affiliations:** 1 UOC Neurologia, Fondazione Policlinico Universitario A. Gemelli IRCCS, Rome, Italy; 2 Dipartimento Di Neuroscienze, Università Cattolica del Sacro Cuore, Rome, Italy

**Keywords:** Stroke, Anton syndrome, MRI, Basilar artery

An 83-year-oldQuery man with a history of hypertension and dyslipidemia presented with visual deficits and dysarthria. A general physical examination was unremarkable. The neurological examination showed severe dysarthria. Although the pupillary light reflex and extraocular movements were intact, there was a lack of eye contact and loss of menace reflex. A visual field defect in all quadrants was observed by confrontation perimetry. Nonetheless, the patient claimed that he was capable of seeing and he was confabulating about his condition.

**Fig. 1:**
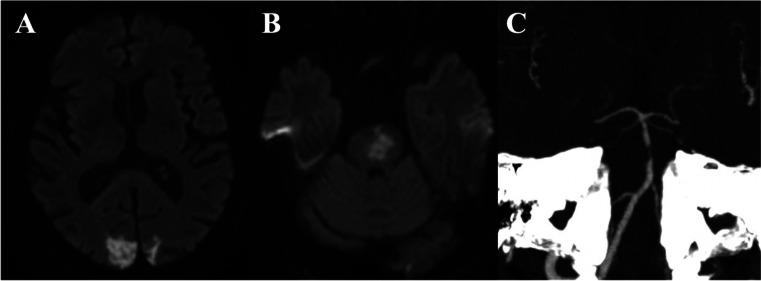
Brain magnetic resonance imaging showed recent ischemic lesions in the occipital lobes bilaterally (panel **A**, diffusion-weighted sequences) and in the pons (panel **B**, diffusion-weighted sequences). Head computed tomography angiography showed a stenosis of the middle third of the basilar artery (panel **C**)

Brain MRI demonstrated acute ischemic lesions in both occipital lobes and in the pons (Fig. 1, panels A and B). CT angiography showed focal stenosis of the basilar artery (Fig. 1, panel C).

Prolonged electrocardiographic monitoring and transthoracic echocardiography were unremarkable. Therapy with acetylsalicylate and statin was started.

Our patient had bilateral occipital ischemic lesions causing cortical blindness and visual anosognosia, while the pontine lesion explained dysarthria. A suspicion of Anton’s syndrome was raised, which is a rare syndrome characterized by blindness without self-awareness of it [1, 2]. The diagnosis of disorders of higher cortical visual function may represent a significant clinical challenge.
